# Personalized Management Strategies in Traumatic Spinal Cord Injury: From Resuscitation to Definitive Surgical Care

**DOI:** 10.3390/jcm15207923

**Published:** 2026-10-14

**Authors:** Peem Sarasombath, Patawut Bovonratwet, Chaisiri Chaichankul, Roongrath Chitragran, Pawin Gajaseni, Chaiyapruk Pundee, Wongthawat Liawrungrueang, Oluwafemi P. Owodunni, Pakawat Chongsathidkiet, Torphong Bunmaprasert

**Affiliations:** 1 Department of Orthopedics, Phramongkutklao Hospital and College of Medicine, Bangkok 10400, Thailand; peems13063@gmail.com (P.S.);; 2 Department of Orthopedic Surgery, Massachusetts General Hospital, Harvard Medical School, Boston, MA 02114, USA; 3 Department of Orthopedics, Samitivej Srinakarin Hospital, Bangkok 10250, Thailand; 4 Department of Emergency Medicine, University of New Mexico School of Medicine, Albuquerque, NM 87106, USA; 5 Department of Neurosurgery, Duke University School of Medicine, Durham, NC 27710, USA; 6 Department of Orthopedics, Faculty of Medicine, Chiang Mai University, Chiang Mai 50200, Thailand

**Keywords:** traumatic spinal cord injury, personalized management, pathophysiology, prehospital, surgical approach

## Abstract

Personalized management of traumatic spinal cord injury (TSCI) is an emerging approach in modern spine and trauma care. Unlike traditional protocol-driven approaches that apply uniform resuscitation, imaging, and surgical strategies across heterogeneous injury patterns, individualized care pathways that account for biological, physiological, and injury-specific variability may improve neurological outcomes and resource utilization. This review summarizes contemporary concepts in personalized TSCI management spanning prehospital and initial hospital care, resuscitation, imaging, and definitive surgical decision-making. The proposed framework is informed by evidence from clinical trials, observational studies, and emerging consensus statements, emphasizing stratified approaches based on neurological status, injury mechanism, physiological reserve, and imaging biomarkers. Selective spinal motion restriction, hemodynamic support within guideline ranges, and early decompression are supported by current guidelines, albeit based largely on low-quality evidence, whereas individualized hemodynamic targets, advanced neuroimaging, blood-based biomarkers, and artificial intelligence-based risk stratification remain emerging strategies that require prospective validation before routine clinical use. In conclusion, personalized management across the continuum of TSCI care provides a rational framework with potential clinical applications, although its effect on patient outcomes remains to be demonstrated.

## 1. Introduction 

Traumatic spinal cord injury (TSCI) represents a major global health burden, resulting in substantial mortality, lifelong disability, and socioeconomic impact [1,2]. Despite advances in trauma systems, surgical techniques, and intensive care, neurological recovery after TSCI remains highly variable and often unpredictable. Traditional management strategies have largely relied on standardized protocols that assume homogeneity across patients, overlooking the profound biological, anatomical, and physiological differences that influence outcomes [3]. The incidence is influenced by age and sex and has remained relatively stable in the United States at approximately 54 cases per million annually. Incidence has declined in younger populations but increased among older adults, with males accounting for nearly 80% of cases. Injury mechanisms vary by age, with falls predominating in the elderly and motor vehicle accidents and firearm injuries most common in younger individuals. Cervical injuries account for approximately half of TSCIs. TSCI imposes a substantial burden, with prolonged hospitalization, frequent rehospitalization, reduced life expectancy, and the highest mortality occurring within the first year after injury [4,5,6]. Despite numerous published guidelines, there is no clear consensus on comprehensive management strategies or effective methods to enhance patients’ quality of life [7,8]. Globally, an estimated 0.9 million new cases of spinal cord injury occurred in 2019, and 20.6 million people were living with the condition, with falls and road injuries as the leading causes [5,9]. The economic burden is substantial: in the United States, average first-year health care and living expenses range from approximately USD 460,000 for motor-incomplete (AIS D) injuries to USD 1.4 million for high tetraplegia, and estimated lifetime costs for a person injured at 25 years of age range from approximately USD 2.1 million to USD 6.3 million (2024 US dollars), excluding indirect costs such as lost productivity [10].

The “one-size-fits-all” approach has increasingly been challenged by emerging evidence demonstrating heterogeneous injury mechanisms and recovery trajectories, underscoring the need for approaches that tailor management to individual patient characteristics, injury biology, and physiological responses [11]. This review aims to synthesize current evidence supporting individualized management of TSCI across the continuum of care, from prehospital assessment to definitive surgical management, while distinguishing established practice from emerging precision tools, biomarkers, and data-driven strategies that still require validation. The central premise of this review, which we frame as a working hypothesis, is that the heterogeneity of TSCI is sufficiently large, and sufficiently identifiable during the acute phase from neurological, mechanistic, physiological, and imaging characteristics, that stratifying patients into clinically recognizable phenotypes could guide acute management decisions more precisely than uniform protocols. Because this review does not generate new data, this hypothesis is not tested here; rather, the proposed framework (Section 7) is intended to make it testable in prospective studies.

Prior reviews have addressed personalized or precision approaches to TSCI within single domains, prehospital resuscitation and hemodynamic management, neuroimaging and biomarker-based prognostication, or surgical timing, but, to our knowledge, none has integrated these domains into a single decision framework spanning the full continuum of care. This review addresses that gap in two ways. First, we propose an integrated, phenotype-driven decision framework (Section 7) that links patient- and injury-level characteristics identifiable at each stage of care, prehospital assessment, hemodynamic resuscitation, imaging, and surgical decision-making, to specific, actionable management pathways, rather than treating each domain in isolation. Second, we provide a structured, domain-by-domain critical gap analysis (Section 8) that translates the evidence limitations discussed throughout this review into specific, testable research priorities, including the largely unaddressed question of how personalized TSCI care pathways can be implemented in resource-variable health systems.

### Method

This perspective review combines a structured literature search with the authors’ multidisciplinary clinical expertise in orthopedic spine surgery, neurosurgery, and emergency and trauma care. PubMed, Scopus, Web of Science, and Google Scholar were searched for articles published between January 2000 and April 2026 using combinations of the terms “spinal cord injury”, “traumatic”, “personalized”, “individualized”, “precision”, “prehospital”, “immobilization”, “airway”, “mean arterial pressure”, “spinal cord perfusion pressure”, “vasopressor”, “magnetic resonance imaging”, “biomarker”, “artificial intelligence”, “machine learning”, “corticosteroid”, “neuroprotection”, “decompression”, and “surgical timing”. The final search was performed on 28 April 2026. Reference lists of key articles and guidelines were hand-searched for additional sources. Articles were eligible if they addressed the acute management of TSCI in humans and reported data or recommendations relevant to patient-level variability or individualized decision-making. Clinical practice guidelines randomized controlled trials, systematic reviews and meta-analyses, and large observational cohorts were prioritized; preclinical studies were included only where they informed injury mechanisms or therapeutic rationale. Non-English-language articles, case reports, conference abstracts, and studies of non-traumatic spinal cord injury were excluded. Given the heterogeneity of study designs and outcomes, no quantitative pooling or formal risk-of-bias assessment was performed. Evidence was synthesized qualitatively by domain (pathophysiology, prehospital and initial assessment, imaging, medical management, and surgical management), and the strength of guideline recommendations is reported as graded by the issuing bodies where available. Draft syntheses were circulated among all authors and revised iteratively until consensus was reached; the resulting phenotype-based framework (Section 7) therefore represents an expert synthesis rather than a formally validated consensus process.

## 2. Individualized Pathophysiology: Understanding Biological and Clinical Variability

TSCI is not a single entity, but a spectrum of injuries shaped by interindividual variability. The primary mechanical injury depends on energy transfer, spinal level, canal diameter, and fracture morphology, and secondary cascades, including ischemia, inflammation, excitotoxicity, and oxidative stress, also vary widely between patients [12]. The spinal cord comprises longitudinal white matter tracts and segmental gray matter that coordinate motor, sensory, and autonomic function. Descending pyramidal and extrapyramidal pathways mediate voluntary movement, posture, and locomotion, while ascending tracts carry pain, temperature, proprioceptive, and vibration information [13]. Autonomic control arises from sympathetic preganglionic neurons in the thoracolumbar cord and parasympathetic neurons in the sacral segments [14].

Neuronal function depends on interactions with astrocytes, microglia, vasculature, and the blood–spinal cord barrier, supported by a regulated extracellular matrix [15,16]. TSCI disrupts these neural–glial–vascular interactions, causing scarring and fibrotic matrix remodeling that underlie the heterogeneity in neurological deficits, autonomic dysfunction, and recovery potential [17].

Acute spinal cord injury has traditionally been conceptualized as an irreversible primary mechanical insult followed by secondary, progressive injury processes. While the primary injury cannot be modified, secondary damage may be mitigated or exacerbated by subsequent factors [18].

Primary traumatic spinal cord injury results from mechanical forces that exceed the tissue’s tolerance, causing immediate structural damage that initiates secondary injury cascades. These forces can produce contusion, stretching, or laceration of the spinal cord, nerve roots, vasculature, and protective barriers, leading to instantaneous axonal and myelin disruption and blood–brain barrier compromise [19]. Injury severity depends on the magnitude, velocity, and type of force applied, ranging from high-energy vehicular trauma and penetrating injuries to low-energy compression in elderly individuals with pre-existing spinal stenosis. Importantly, even in the absence of fracture, rapid cord deformation can result in severe neurological injury, highlighting the biomechanical vulnerability of the spinal cord and the heterogeneity of primary injury patterns [20].

Secondary injury is a time-dependent cascade that amplifies the initial mechanical insult [21,22]. Its principal components, namely calcium-mediated mitochondrial dysfunction and oxidative stress, ischemia–reperfusion injury driven by vascular disruption and neurogenic shock, glutamate excitotoxicity, and a multicellular inflammatory response, begin within minutes, peak over days, and extend into chronic demyelination and neurodegeneration. From a personalized-management perspective, the relevance of these mechanisms lies in their variability among patients: the extent of cord ischemia depends on systemic blood pressure, the severity of neurogenic shock, and pre-existing vascular disease; the inflammatory response is modulated by age, comorbidity, and associated injuries; and the time course of secondary injury differs between individuals. This variability provides the biological rationale for individualizing modifiable factors such as perfusion targets, the timing of decompression, and eligibility for neuroprotective trials [23,24,25,26,27,28,29].

Because secondary injury cannot be directly observed at the bedside, surrogate markers are needed to capture this interindividual variability. MRI features such as intramedullary hemorrhage, edema length, and degree of cord compression provide prognostic information beyond clinical examination alone, and higher serum concentrations of neural and glial injury biomarkers have been associated with greater injury severity and worse clinical outcomes. These imaging and fluid biomarkers, discussed further in Section 4, are candidate tools for stratifying patients according to biological injury severity rather than clinical grade alone [30,31].

Genetic predisposition, metabolic status, age, comorbidities, and baseline vascular health further influence neurological recovery. Collectively, these factors explain why TSCI patients require individualized treatment pathways, rather than uniform protocols applied indiscriminately [32,33].

## 3. Precision Prehospital and Initial Hospital Assessment

The primary objective of prehospital and initial hospital spinal cord injury management is to minimize neurological damage and prevent secondary deterioration. This requires rapid on-scene assessment, immediate stabilization of airway, breathing, and circulation, followed by focused secondary evaluation and prompt transport to an appropriate trauma center for definitive care [34]. Personalized risk assessment based on mechanism of injury, patient age, and comorbidities allows for selective spinal motion restriction, moving away from universal immobilization strategies that may cause harm in low-risk patients [35].

### 3.1. Airway Management

Airway and ventilation decisions should account for neurological level, respiratory reserves, level of consciousness, and associated injuries, while cervical spine motion is minimized by cervical spine stabilization [36]. A rapid assessment is performed to determine whether the patient has a Glasgow Coma Scale (GCS) score of 8 or less [37]. In such cases, published protocols generally describe preoxygenation, manual in-line stabilization, intubation by an experienced operator using the laryngoscopy technique and adjuncts with which they are most proficient, a prepared difficult-airway strategy, and subsequent lung-protective ventilation with frequent reassessment. These steps largely reflect general trauma airway principles; evidence specific to TSCI is limited, and some components, such as cricoid pressure, remain debated. Airway strategies should therefore be adapted to the individual patient, operator experience, and local resources rather than applied as a fixed sequence [38,39,40,41,42].

### 3.2. Hemodynamic Management

Hemodynamic resuscitation in acute TSCI aims to prevent secondary ischemic injury [43]. The 2013 AANS/CNS guidelines recommended maintaining a mean arterial pressure (MAP) of 85–90 mmHg for the first 7 days after injury. The more recent 2024 AO Spine/Praxis guideline suggests augmenting MAP to a lower limit of at least 75–80 mmHg without active augmentation beyond an upper limit of 90–95 mmHg, for a duration of 3–7 days; both recommendations are weak [44]. These thresholds are therefore best regarded as pragmatic ranges rather than validated targets. Several observational studies suggest that fixed targets may not account for interindividual variability in spinal cord perfusion and physiological reserve. In an analysis of high-frequency physiological data, higher MAP was associated with motor recovery predominantly during the first days after injury, and a topological network analysis identified a non-linear MAP range of approximately 76–104 mmHg, deviation from which was associated with poorer neurological outcomes. Small prospective and retrospective series have not shown a clear increase in major cardiovascular adverse events, such as hemorrhage, stroke, or myocardial infarction, when MAP was maintained above 85 mmHg for 3–7 days [45,46,47,48,49]. Spinal cord perfusion pressure (SCPP), measured with intrathecal or intraparenchymal monitoring, has been reported to correlate more closely with neurological recovery than MAP alone, with optimal thresholds varying between patients and over time. Because these data are observational and derived from relatively small cohorts, they demonstrate associations rather than causal effects and do not yet support any specific individualized target; they do, however, provide the rationale for prospective evaluation of perfusion-guided, individualized hemodynamic management [50,51].

Vasopressor selection must be tailored to individual cardiovascular profiles, while fluid resuscitation strategies should balance spinal perfusion against risks of edema and pulmonary complications. Temperature control, glucose management, and metabolic optimization further require individualized thresholds. In acute SCI, vasopressors mainly increase systemic vascular resistance and MAP, whereas inotropes improve cardiac output [52]. Norepinephrine is commonly used as a first-line vasopressor in acute SCI because it effectively increases blood pressure and spinal cord perfusion with relatively favorable physiologic effects [53]. However, it should be used cautiously in patients with peripheral vascular disease to avoid worsening ischemia. Prolonged administration also requires central venous access, as peripheral infusion carries a risk of local tissue injury and necrosis [54,55,56]. Dopamine is less favored due to its association with increased intrathecal pressure, reduced spinal cord perfusion, and higher rates of arrhythmias and myocardial injury [57]. Epinephrine provides strong cardiovascular support but carries significant risks and is reserved for refractory hypotension [58]. Phenylephrine increases vascular resistance without affecting heart rate, making it useful in selected patients, though it may be inferior to norepinephrine for spinal cord perfusion [59]. Dobutamine primarily improves cardiac output and is best used in patients with myocardial dysfunction, often in combination with vasopressors [60]. However, vasopressor use has been associated with complications beyond cardiovascular effects, including higher rates of infection and procedural adverse events such as pneumonia, deep vein thrombosis, unplanned reoperations, and surgical site infections, although these observational associations may partly reflect greater underlying injury severity [46].

### 3.3. Immobilization and Transport

Early identification of high-risk patients enables rapid triage and direct transport to specialized spinal trauma centers, minimizing delays in definitive care. A survey reported few respondents (12.3%) used American Association of Neurological Surgeons (AANS) guidelines for prehospital immobilization and transport in acute TSCI [61], while most relied on locally developed protocols (61.5%). In adults and children older than 12 years, rigid spine boards can cause discomfort. Padding the spine board improves patient comfort and reduces pressure at the occipital and sacral regions without diminishing biomechanical stability [62]. Selective immobilization algorithms illustrate how risk stratification can be applied at the scene. One such algorithm for adults (>16 years) first screens for high-risk clinical features (intoxication, altered consciousness, spinal pain, neurological deficit, or significant pre-existing spinal disease), then for a dangerous mechanism of injury, and then for abnormal neurology, midline spinal tenderness, or distracting injury; patients without these features who can actively rotate the neck 45° to the left and right and mobilize without pain or neurological symptoms are considered low risk and may not require immobilization [63]. Such rules have been evaluated mainly in alert adults within well-resourced emergency medical systems and should be applied within local protocols rather than as universal prescriptions.

Direct transfer to spinal cord injury units was most commonly limited by associated injuries, restrictive policies, and geographic distance, with fewer respondents citing aeromedical transport or bed availability. Only about one-third reported the presence of a non-refusal spinal cord injury unit admission policy in their region [64].

## 4. Imaging and Diagnostic Pathways

Imaging modalities play complementary roles in TSCI evaluation: plain radiographs remain part of routine trauma imaging but are less sensitive for subtle fractures and soft tissue pathology. CT is highly sensitive for detecting fractures and dislocations, whereas MRI is more effective for assessing soft tissue injury, spinal cord disruption, and edema [65,66]. In addition, the 2019 American Spinal Injury Association (ASIA) classification is used to diagnose and grade SCI according to injury completeness and the zone of partial preservation [67]. In acute TSCI, MRI shows injuries ranging from cord concussion to compression, edema, hemorrhage, and transection, with greater compression, longer edema, and intramedullary hemorrhage predicting worse outcomes. Key sequences include T1/T2, GRE for hemorrhage, and DWI for detecting ischemia or occult injury. In the subacute phase (2–6 weeks), MRI is crucial for evaluating neurological deterioration and identifying complications such as ascending myelopathy or early syrinx while excluding other causes [68]. However, this approach has been criticized, as despite all guidelines recommending MRI for patients with suspected TSCI or spinal cord injury without radiographic abnormality (SCIWORA), covering patient age, timing, clinical indications, and MRI techniques, the supporting evidence remains predominantly low quality, leading to recommendations that are weak and not strongly evidence based [69]. Imaging strategies should be adapted to patient stability, injury mechanism, and clinical priorities.

In the acute setting, multidetector CT is the first-line imaging modality for suspected spinal column injury in adult trauma patients because it is rapid, widely available, and highly sensitive for fractures and malalignment, and it has largely replaced plain radiographs as the initial screening test where available [70]. MRI is the most sensitive and accurate modality for evaluating the spinal cord itself, including cord compression, edema, hemorrhage, and disk and ligamentous injury, and guidelines suggest obtaining MRI before surgical intervention, when feasible, to facilitate surgical decision-making [71]. Ultrasound has no established role in the primary diagnosis of TSCI in adults because the bony spinal canal limits the acoustic window; its main application is intraoperative, after laminectomy, when it allows real-time assessment of the adequacy of decompression and of cord swelling, although it is currently used by a minority of spine surgeons [72].

Advanced quantitative MRI techniques detect microstructural and secondary injury changes across the neuraxis that are not visible on standard imaging. Importantly, MRI biomarkers obtained early after injury have been associated with functional recovery and, when combined with clinical or biological markers, may improve prognostic accuracy. Together, conventional and quantitative MRI hold promise as neuroimaging biomarkers to guide personalized care and serve as surrogate endpoints for future acute and chronic SCI clinical trials [73,74]. Biomarker-driven timing of MRI may optimize resource utilization and clinical relevance. Advanced imaging techniques such as diffusion tensor imaging (DTI) and functional MRI hold promise for personalized prognostication. Integrating imaging with clinical and laboratory data may enable decision-support models for individualized care planning.

Artificial intelligence (AI) has been explored for both diagnosis and prognosis in TSCI. General-purpose large language models have been evaluated against spine-injury management guidelines using guideline-based clinical questions; although their responses broadly aligned with best-practice recommendations, such performance does not establish safety or effectiveness in real-world trauma settings, and these models are currently best regarded as research tools [75,76,77]. Of greater relevance to personalized management are prediction models trained on clinical and imaging data. In a recent meta-analysis, machine-learning models predicted outcomes after spinal cord injury with reasonable accuracy, particularly for functional prognosis, with gradient-boosting (XGBoost) models performing best [78]; however, these studies report prognostic discrimination rather than clinical utility or improvements in patient outcomes. Most models were developed on retrospective datasets, and their calibration, generalizability, clinical utility, and safety require prospective external validation before routine clinical use. AI should therefore be regarded as an emerging research opportunity rather than an established component of personalized TSCI management.

Therefore, imaging-derived information can contribute to patient stratification at several points in the care pathway: (i) identifying ongoing cord compression that may warrant urgent decompression, as opposed to non-compressive or SCIWORA-type injuries; (ii) estimating biological injury severity (e.g., intramedullary hemorrhage and lesion length) to refine prognosis beyond the AIS grade, which may inform counseling, rehabilitation planning, and clinical-trial eligibility; (iii) distinguishing acute injury from pre-existing degenerative compression in older adults; and (iv) re-evaluating patients with early neurological deterioration, in whom the clinical trajectory combined with repeat imaging can inform the urgency of intervention. Structured algorithms incorporating these patient-specific data may help differentiate surgical emergencies from candidates for close observation, although such algorithms have not yet been prospectively validated. These applications underpin the imaging pathways proposed for each phenotype in Table 1.

## 5. Personalized Management

Within the hospital setting, individualized trauma profiles should be integrated into Advanced Trauma Life Support (ATLS) principles [79]. Neurological assessment using ASIA/AIS grading must be interpreted in the context of injury pattern, imaging findings, and physiological stability [80].

For individualized decision-making, acute therapies can be grouped into three categories according to the strength of the supporting evidence (Table 2): (i) guideline-supported standard care; (ii) optional or controversial treatments, whose use depends on clinical context and patient-specific risk–benefit assessment; and (iii) experimental therapies, which should currently be offered only within clinical trials. At present, no pharmacological or biological therapy has definitive evidence of meaningful neurological benefit in TSCI. Most investigated agents aim to mitigate secondary injury mechanisms, such as inflammation, excitotoxicity, oxidative stress, and immune dysregulation, rather than directly restore neural function. Non-steroidal anti-inflammatory drugs (NSAIDs), monosialotetrahexosylganglioside (GM-1) ganglioside, growth factors, minocycline, Rho/ROCK inhibitors, and antibody-based therapies have shown encouraging results in preclinical models or selected patient subgroups, but clinical trial results have been inconsistent and insufficient to support routine use [81,82,83,84,85]. Riluzole was safe in a phase III trial but did not meet its primary efficacy endpoint in acute cervical SCI; secondary analyses suggested improved neurological and functional recovery, particularly in AIS B–C patients, but these findings should be regarded as hypothesis-generating [86]. Regenerative strategies, including mesenchymal stem cell–based therapies and extracellular vesicle–based delivery systems, are among the most actively investigated approaches; however, the supporting evidence is preliminary, derived largely from preclinical and small early-phase clinical studies with methodological limitations, and these therapies remain experimental.

The role of corticosteroids remains controversial and should be guided by patient-specific risk–benefit assessment rather than routine administration (Table 2). Although the National Acute Spinal Cord Injury Study (NASCIS) II and III briefly established methylprednisolone sodium succinate (MPSS) as standard therapy for acute SCI, these trials were heavily criticized for methodological limitations and were associated with significant adverse events [87,88]. Accumulating evidence since the early 2000s has led most major guidelines to recommend against steroid use in SCI, whereas the 2017 AO Spine guideline lists a 24 h MPSS infusion started within 8 h of injury as a treatment option [89,90,91]. Multiple meta-analyses have since shown no meaningful neurological benefit from high-dose MPSS, even when given early, and although global use has declined, steroids remain in use in some regions due to persistent beliefs in efficacy and medicolegal concerns [92]. In a global AOSpine survey, ~53% of spine surgeons still used methylprednisolone for acute SCI, most commonly in Asia-Pacific, Middle East, and Latin America, often based on NASCIS III, with lower use among higher-volume surgeons, highlighting regional gaps [93]. At North American Clinical Trials Network centers, steroid use declined sharply after 2009 and was associated with higher pulmonary complication rates, underscoring ongoing controversy and the need for individualized decision-making [94]. An updated meta-analysis found no short- or long-term neurological benefit. In a retrospective cohort of cervical SCI patients treated with modified-dose methylprednisolone, complications were mainly ICU-related (pneumonia, anemia, dysphagia), while gastrointestinal hemorrhage was uncommon and associated only with transfusion, suggesting lower-than-expected GI risk with modified dosing [95,96,97]. Therefore, these data do not support routine use; where MPSS is considered, the decision should be made on an individual basis with explicit discussion of the uncertain benefit and the risk of complications.

**Table 2:** Evidence-based classification of acute management options in traumatic spinal cord injury.

Category	Intervention	Current Evidence and Considerations for Individualization
Guideline-supported standard care	Early surgical decompression (<24 h when medically feasible)	Recommended in the 2024 AO Spine/Praxis update on the basis of moderate-quality evidence; benefit of ultra-early (<12 h) surgery uncertain; timing within the window depends on physiological stability and polytrauma burden [98,99]
Guideline-supported standard care	MAP augmentation (lower limit ≥ 75–80 mmHg; upper limit 90–95 mmHg) for 3–7 days	Weak recommendation based on very low-quality evidence; optimal target and duration by phenotype unknown [44]
Guideline-supported standard care	ATLS-based resuscitation and selective spinal motion restriction	Standard of care; selective immobilization criteria evaluated mainly in alert adults in well-resourced EMS systems [63,73]
Optional or controversial	High-dose MPSS (24 h infusion started within 8 h)	Listed as a treatment option by the 2017 AO Spine guideline but not recommended by most other guidelines; meta-analyses show no consistent neurological benefit and an increased complication risk [92]
Optional or controversial	SCPP-guided hemodynamic management	Associated with neurological recovery in observational cohorts; requires invasive monitoring; no guideline recommendation [44]
Experimental	Riluzole	Phase III trial did not meet its primary endpoint; secondary analyses are hypothesis-generating [86]
Experimental	Other neuroprotective agents (e.g., GM-1, minocycline, Rho/ROCK inhibitors, antibody-based therapies)	Preclinical or early-phase evidence with inconsistent clinical results; use within clinical trials only [81,82,83,84,85]
Experimental	Cell-based and extracellular vesicle–based therapies	Preliminary preclinical and early-phase clinical evidence; use within clinical trials only
Experimental	AI-based prediction and decision support	Promising retrospective performance; lacks prospective validation and decision-impact data [78]

## 6. Tailored Surgical Management

Current guidelines for the surgical management of traumatic spinal cord injury emphasize early stabilization and mitigation of secondary injury while acknowledging persistent evidence gaps. Urgent closed reduction of cervical fracture–dislocation injuries in awake, examinable patients is considered safe and effective, with a low risk of permanent neurological deterioration, and routine prereduction MRI is not recommended in this setting because it may delay realignment and decompression.

### 6.1. Timing of Surgery (Early vs. Delayed)

Current evidence supports early surgical decompression after traumatic spinal cord injury (SCI) to improve neurological recovery. A 2024 clinical practice guideline update recommends that adult patients with acute SCI undergo decompressive surgery within 24 h of injury when medically feasible, based on moderate evidence that early decompression increases the likelihood of meaningful improvements in ASIA Impairment Score and motor recovery compared with later intervention, although evidence for ultra-early surgery (<12 h) remains inconsistent due to small sample sizes and variable definitions [98]. A separate multinational survey of spinal surgeons found variation in real-world practice: most Latin American surgeons reported performing early (<24 h) surgery more commonly for incomplete injuries and traumatic cauda equina syndrome than for complete SCI or central cord syndrome, with resource limitations such as implant availability and delayed transfers cited as barriers to early surgery [99]. In a survey conducted in the Netherlands, surgeons generally prioritize earlier surgery for incomplete TSCI compared with complete injuries. Despite guidelines recommending decompression within 24 h, approximately 43% of patients with complete TSCI are not operated upon within this timeframe. Although traumatic central cord injury is the most common incomplete subtype, it is typically managed less urgently than other incomplete TSCI patterns, highlighting that initial neurological severity strongly influences surgical timing [100].

Overall, within the guideline-recommended window, the precise timing of surgery may be individualized according to neurological status, hemodynamic stability, and polytrauma burden. Some patients may require physiological optimization before surgery, and the optimal timing for older adults and for patients with central cord syndrome remains less certain. Personalization in this context concerns the feasibility, safety, and clinical context of early surgery, rather than implying that deliberate delay is beneficial for any phenotype.

### 6.2. Individualized Surgical Approaches

Surgical strategy must reflect spinal region, fracture morphology, bone quality, and patient comorbidities. Decision-making in TSCI requires more than assessing spinal cord compression and neurological deficit; factors such as injury mechanism, biomechanical stability, osteo-ligamentous integrity, and fracture morphology are also critical. Earlier classification systems have not been widely adopted because they are complex [101,102,103,104], insufficiently incorporate modern imaging such as MRI, and do not clearly guide operative versus conservative management or account for neurological injury patterns. Subsequently, the Spine Trauma Study Group introduced the Subaxial Injury Classification (SLIC) system for subaxial cervical spine injuries and the Thoracolumbar Injury Classification and Severity Score (TLICS) [105,106]. These classification systems have been extensively discussed in the literature and are widely accepted as valuable tools for initial surgical decision-making, as they systematically incorporate injury morphology, ligamentous integrity, and neurological status into a structured and reproducible framework. Nevertheless, important limitations persist, including dependence on subjective clinician interpretation, uncertainty in cases with intermediate scores, and limited ability to account for patient-specific factors, dynamic spinal instability, and evolving neurological deficits. Consequently, definitive management decisions must continue to rely on individualized clinical judgment rather than classification scores alone [107,108].

Decisions regarding the anterior, posterior, or combined surgical approach in the management of acute TSCI should be dictated by the specific location and mechanism of compression, fracture morphology, and biomechanical instability, rather than a universal preference for any single technique. Anterior approaches are typically favored for relieving anterior mass effect from disk herniation or vertebral body fragments, while posterior approaches facilitate direct posterior decompression and indirect expansion of the spinal canal and are often preferred for posterior element injuries or facet dislocations. Combined anterior–posterior surgery may be indicated in cases of severe multicolumn instability or when neither anterior nor posterior access alone can achieve sufficient decompression and stabilization. Contemporary recommendations underscore that no single approach has consistently demonstrated superior neurological outcomes across all injury patterns, and adequate decompression, sometimes achieved through adjuncts such as laminectomy or intraoperative ultrasound, remains the primary objective, with the technique selected based on individual injury characteristics and goals of care [109,110].

For acute cervical spinal cord injury, anterior decompression is preferred for dominant ventral compression or disk herniation and is generally less invasive, but anterior-only fixation carries a risk of failure or graft migration in unstable injuries, often necessitating secondary posterior surgery [111,112]. Posterior approaches provide stronger stabilization for posterior element injuries and facet dislocations but are associated with specific risks, including vertebral artery injury and unresolved controversy regarding fixation across the cervicothoracic junction, balancing hardware failure against increased surgical morbidity [113,114]. Combined approaches may be indicated in selected cases with severe multicolumn instability or compromised anterior column integrity but are associated with higher operative complexity and morbidity [115].

In acute thoracolumbar spinal cord injury, fracture–dislocations are the most common and severe injury pattern and typically require urgent posterior decompression and pedicle screw fixation once the patient is medically stabilized [116]. The posterior approach is favored due to safer anatomy, superior stability and visualization, and greater surgeon familiarity, while current evidence shows no consistent neurological advantage of anterior over posterior approaches [117]. Isolated anterior surgery may be considered in carefully selected patients without neurological deficits, but its role in high-energy injuries remains unproven [118,119]. Individualized surgical planning should also consider the biomechanical consequences of the construct. In adult spinal deformity surgery, long fusions to S1 with or without pelvic fixation have been shown to induce acute changes in pelvic incidence, a parameter traditionally regarded as fixed [120]. Although these findings cannot be directly extrapolated to acute TSCI, they illustrate that construct length and pelvic fixation can alter spinopelvic alignment and should be considered when instrumentation must be extended to the lumbosacral junction.

Overall, no approach has demonstrated consistent superiority in neurological outcomes; thus, timely and adequate decompression remains the primary goal, with surgical strategy guided by individual injury characteristics and clinical context rather than a fixed algorithm. Decisions regarding anterior, posterior, or combined approaches should be personalized to maximize stability, decompression, and recovery while minimizing morbidity.

## 7. Toward a Personalized TSCI Care Pathway: An Integrated Decision Framework

**Figure 1:**
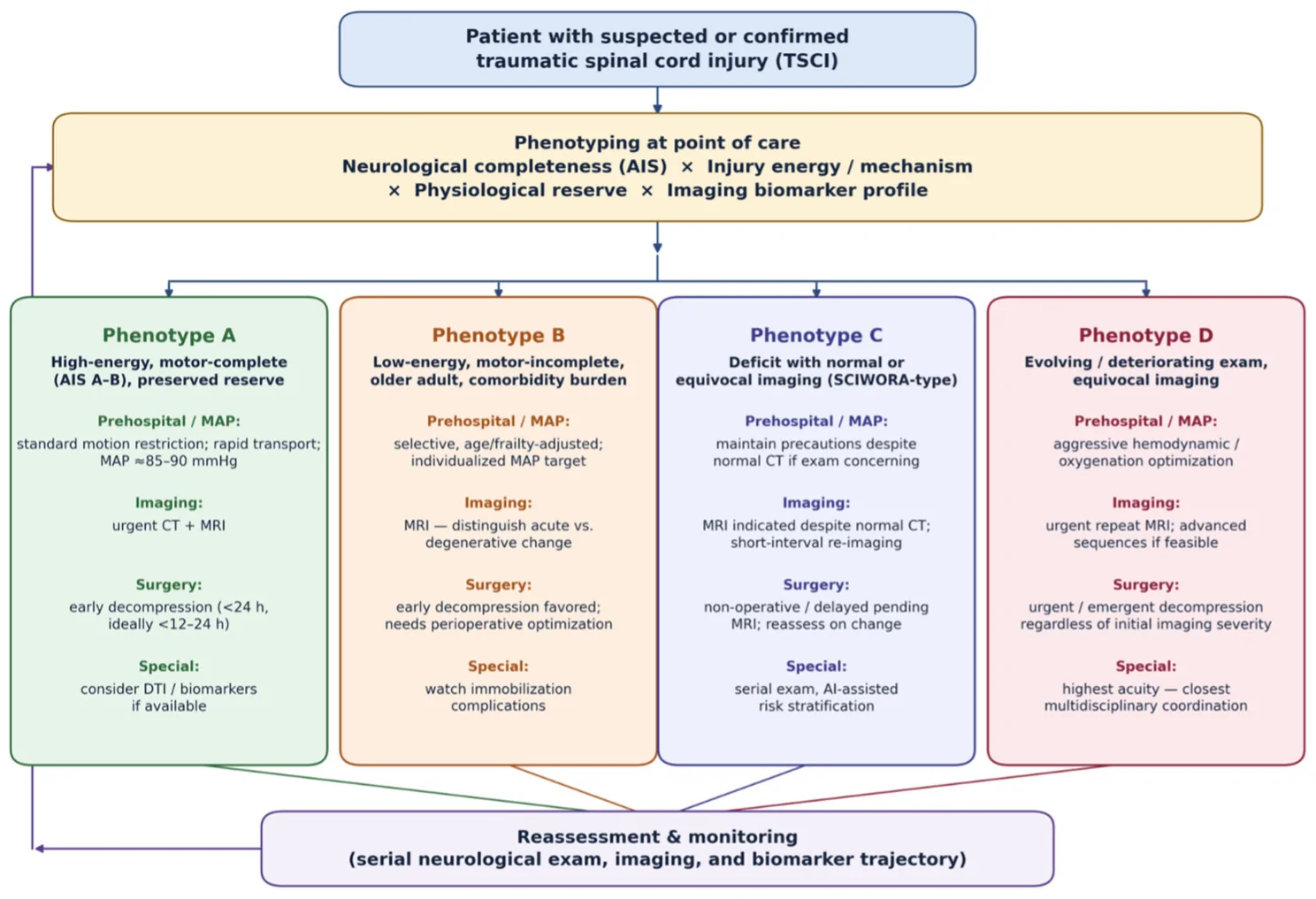
Personalized traumatic spinal cord injury (TSCI) care pathway: an integrated, phenotype-driven decision framework. Patients are phenotyped at the point of care by neurological completeness, injury energy/mechanism, physiological reserve, and imaging biomarker profile, yielding four representative phenotypes (A–D) that map to differentiated prehospital/hemodynamic, imaging, and surgical-timing pathways (detailed in Table 1), followed by continuous reassessment and re-phenotyping as the clinical trajectory evolves.

The preceding sections illustrate that individual elements of TSCI care, such as hemodynamic targets, imaging strategy, and surgical timing, have each been studied largely as isolated decision points. In clinical practice, however, these decisions are made sequentially and interdependently for the same patient. We propose organizing this continuum around four representative injury phenotypes, defined by neurological completeness, injury energy/mechanism, physiological reserve, and imaging biomarker profile (Table 1, Figure 1). Briefly, Phenotype A comprises high-energy, motor-complete injuries (AIS A–B) in patients with preserved physiological reserves; Phenotype B comprises low-energy, motor-incomplete injuries in older adults with comorbidities and pre-existing degenerative stenosis, typically presenting as a central cord pattern; Phenotype C comprises neurological deficits with normal or equivocal initial radiographs or CT (SCIWORA-type presentations); and Phenotype D comprises patients whose neurological examination is evolving or deteriorating, irrespective of initial imaging findings. The defining features of each phenotype and the corresponding management considerations are provided in Table 1. This phenotype-to-pathway framework is intended as a synthesis and organizing scaffold for clinical reasoning and future prospective validation, rather than a validated clinical decision rule.

This framework highlights that “personalization” in TSCI care is not a single axis (e.g., injury severity alone) but the intersection of neurological, mechanistic, physiological, and imaging variables, and that the same evidence reviewed in isolation throughout Section 2, Section 3, Section 4, Section 5 and Section 6 yields different management implications depending on which phenotype a patient falls into. Figure 1 renders this framework as a decision flow diagram, integrating phenotyping, domain-specific actions, and a reassessment loop that reflects the dynamic nature of TSCI trajectories.

The recommendations in Table 1 differ in their evidentiary status. Elements that correspond to current guideline recommendations for TSCI in general, such as MAP augmentation within guideline ranges, early decompression within 24 h when medically feasible, and MRI for patients with neurological deficits or SCIWORA-type presentations, are applied to each phenotype, but none have been tested in comparative studies within the phenotypes defined here. Phenotype-specific adaptations, such as frailty-adjusted motion restriction and individualized MAP targets in Phenotype B, structured serial neurological monitoring in Phenotype C, and prioritizing the clinical trajectory over static imaging in Phenotype D, are extrapolations from the broader TSCI literature and represent proposed strategies that require prospective validation. The four phenotypes were selected because they correspond to presentations commonly encountered in clinical practice in which the evidence reviewed in Section 3, Section 4, Section 5 and Section 6 implies different management priorities; they are not intended to be exhaustive or mutually exclusive. The framework should therefore be read as a conceptual model rather than a validated clinical decision algorithm.

### 7.1. From Acute Phenotype to Rehabilitation Planning

Acute phenotyping also informs the transition to multidisciplinary rehabilitation, which should begin as soon as the patient is medically stable [121]. Phenotype-specific needs differ, including ventilator weaning in high cervical complete injuries (Phenotype A), frailty and dysphagia in older adults with central cord syndrome (Phenotype B), and autonomic dysfunction in injuries at or above T6. Care in specialized SCI centers has been associated with shorter length of stay and fewer complications [122]. Importantly, neurological recovery should be distinguished from functional outcomes, such as independence, mobility, and quality of life, measured by tools like the Spinal Cord Independence Measure [123]. Given the limited trial evidence for rehabilitation in spinal disorders [124], future studies of personalized TSCI pathways should include functional and patient-reported outcomes.

### 7.2. Applicability Across Healthcare Settings

The framework can be applied across resource settings. Essential components (ATLS-based resuscitation, selective motion restriction, MAP within guideline ranges, spinal CT, serial neurological examination, and early decompression or transfer when feasible) are achievable in most systems. MRI and specialized SCI units enhance individualization when available, whereas SCPP monitoring, quantitative MRI, blood biomarkers, and AI tools remain investigational and are not prerequisites. Where MRI is unavailable, phenotypes can still be assigned from clinical examination, mechanism, physiology, and CT findings (Table 3).

## 8. Critical Gaps and Research Agenda

Two broad types of gaps currently limit the implementation of personalized TSCI care: technological gaps and gaps in clinical evidence. With respect to technological gaps, the integration of precision medicine into trauma systems will require substantial advances in data harmonization, real-time analytics, advances in imaging or biomarkers and interdisciplinary collaboration across prehospital, surgical, neurocritical care, and rehabilitation domains. Large-scale clinical registries, multimodal monitoring, and interoperable digital platforms will be essential to enable timely synthesis of imaging, physiological, molecular, and clinical data [125,126]. Big data analytics and machine-learning–driven models hold promise for dynamic risk stratification, outcome prediction, and personalized decision support; however, their successful implementation depends on rigorous validation, and continuous clinical oversight [127,128,129]. Gaps in clinical evidence, in contrast, concern whether acting on this information improves patient outcomes. For most of the personalized strategies discussed in this review, including individualized MAP or SCPP targets, biomarker-guided imaging or surgical timing, and phenotype-stratified care pathways, there are no adequately powered prospective studies demonstrating benefit over current standardized care.

Beyond these technological advances, realizing personalized TSCI care requires research specifically designed to test whether phenotype-stratified management, as proposed in Section 7, improves outcomes relative to current uniform protocols. Table 3 summarizes domain-specific evidence limitations identified throughout this review, classifies each as primarily a technological (T) or clinical evidence (C) gap, and translates them into specific, actionable research priorities.

Addressing these gaps will require coordinated, multicenter, and ideally multinational collaboration, given that injury patterns, EMS infrastructure, and imaging/AI resource availability vary substantially across health systems.

## 9. Conclusions

Personalized management of TSCI is an emerging approach that seeks to complement protocol-driven care with biologically and physiologically informed, patient-centered decision-making. Several of its components, such as selective spinal motion restriction, hemodynamic support within guideline ranges, and early decompression when medically feasible, are already part of current practice, whereas individualized perfusion targets, imaging and blood-based biomarkers, and AI-based decision support remain investigational. The approach has potential clinical applications, but its effects on neurological outcomes, safety, and resource use have yet to be demonstrated in prospective studies. Because the evidence underlying these conclusions derives largely from observational studies, expert synthesis, and guideline recommendations of low certainty, they should be regarded as preliminary and interpreted with caution, and further research on this topic is warranted.

## Figures and Tables

**Table 1:** Phenotype-to-pathway framework for personalized TSCI management.

Phenotype	Prehospital/Hemodynamic Target	Imaging Pathway	Surgical Timing	Special Considerations
A. High-energy, motor-complete (AIS A–B), preserved physiological reserve	Standard spinal motion restriction per high-risk criteria; rapid transport to an SCI-capable center; MAP augmentation within guideline ranges (lower limit ≥ 75–80 mmHg; upper limit 90–95 mmHg) for 3–7 days, with vasopressor support as needed	Urgent CT for osseous instability; urgent MRI to characterize cord compression/hemorrhage extent	Early decompression (<24 h when medically feasible; additional benefit of <12 h uncertain)	Prioritize for advanced imaging biomarkers/DTI where available, given favorable physiological tolerance for aggressive management
B. Low-energy, motor-incomplete, older adult with comorbidity burden (e.g., central cord pattern, degenerative stenosis)	Selective, age/frailty-adjusted motion restriction; MAP target individualized within guideline ranges and balanced against cardiopulmonary tolerance	MRI to distinguish acute injury from pre-existing degenerative change	Early decompression may be considered, but optimal timing is less certain in this group, and requires closer perioperative and anesthesia/cardiology optimization	Higher risk of immobilization-related complications (pressure injury, dysphagia); needs multidisciplinary comorbidity management
C. Deficit with normal or equivocal initial imaging (SCIWORA-type presentation)	Maintain spinal precautions despite normal CT if exam is concerning	MRI still indicated despite normal radiographs/CT (guideline-recommended despite a low-quality evidence base); short-interval re-imaging if deterioration	Non-operative or delayed pending MRI findings; reassess if the exam changes	Candidate group for structured, serial neurological monitoring; and AI-assisted risk stratification is a potential future application that requires validation
D. Evolving/deteriorating neurological exam, equivocal imaging	Hemodynamic and oxygenation optimization while workup proceeds	Urgent repeat MRI; advanced sequences (DTI) if time and resources permit	Urgent/emergent decompression regardless of initial imaging severity, clinical trajectory outweighs static imaging findings	Highest-acuity group; requires closest multidisciplinary (trauma, spine, critical care) coordination

Guideline-concordant elements are applied to each phenotype by extrapolation from the general TSCI literature; phenotype-specific adaptations are proposed strategies that have not been evaluated in comparative studies and require prospective validation.

**Table 3:** Critical gaps and research priorities for personalized TSCI care.

Domain	Current Limitation	Specific Research Priority
Prehospital triage (C)	Selective spinal motion-restriction criteria are validated mainly in high-income EMS systems; generalizability to systems with longer transport times or non-physician-led triage is unclear	Prospective validation of selective motion-restriction protocols across heterogeneous EMS systems, including resource-variable settings
Hemodynamic resuscitation (C; T for SCPP monitoring)	Guideline MAP ranges (lower limit ≥ 75–80 mmHg; upper limit 90–95 mmHg) rest on very low-quality evidence	Phenotype-stratified prospective trial comparing individualized versus fixed MAP targets, powered for neurological outcome
Neuroimaging biomarkers (T)	Quantitative MRI/DTI biomarkers show prognostic promise but lack standardized acquisition protocols and multicenter validation	Multicenter harmonization and threshold-validation studies linking quantitative imaging biomarkers to functional outcome
AI-based decision support (T and C)	Current models are trained and validated largely on retrospective, homogeneous datasets; real-time and cross-population performance is unproven	Prospective, multi-site external validation with clinical decision-impact endpoints (not accuracy alone), including diverse and lower-resource populations
Surgical timing in incomplete or older-adult injury (C)	Evidence favoring early decompression derives largely from younger, motor-complete cohorts; optimal timing in older, comorbid, or purely incomplete injury is less certain	Age- and comorbidity-stratified prospective cohorts or trials of early versus delayed decompression
Implementation across resource settings (C)	Most personalized-care evidence originates from high-resource trauma systems; feasibility of individualized hemodynamic monitoring, advanced imaging, and AI tools in resource-variable systems (including much of Southeast Asia) is largely unstudied	Implementation-science studies evaluating adapted, resource-appropriate personalized TSCI care pathways

## Data Availability

No new data were created or analyzed in this study. Data sharing is not applicable to this article.

## Abbreviations

The following abbreviations are used in this manuscript:

TSCI	Traumatic spinal cord injury
SCI	spinal cord injury
MRI	Magnetic Resonance Imaging
GCS	Glasgow Coma Scale
MAP	Mean arterial pressure
SCPP	Spinal cord perfusion pressure
AANS	American Association of Neurological Surgeons
ASIA/AIS	American Spinal Injury Association/ASIA Impairment Scale
SCIWORA	Spinal cord injury without radiographic abnormality
DTI	Diffusion tensor imaging
AI	Artificial intelligence
ATLS	Advanced Trauma Life Support
NSAIDs	Non-Steroidal Anti-Inflammatory Drugs
GM-1	Monosialotetrahexosylganglioside
MPSS	Methylprednisolone sodium succinate
NASCIS	the National Acute Spinal Cord Injury Study
ICU	Intensive Care Unit
SLIC	Subaxial Injury Classification
TLICS	Thoracolumbar Injury Classification and Severity Score
